# Associations Between Symptom Complexity and Acute Care Utilization Among Adult Advanced Cancer Patients Followed by a Palliative Care Service

**DOI:** 10.3390/curroncol32070388

**Published:** 2025-07-04

**Authors:** Philip Pranajaya, Vincent Ho, Mengzhu Jiang, Vance Tran, Aynharan Sinnarajah

**Affiliations:** 1 Faculty of Health Sciences, Queen’s University, Kingston, ON K7L 3N6, Canada; philip.pranajaya@mail.utoronto.ca; 2 Division of Palliative Medicine, Department of Medicine, Lakeridge Health, Oshawa, ON L1G 2B9, Canada; vho@lh.ca (V.H.); mjiang@lh.ca (M.J.); vtran@lh.ca (V.T.); 3 Department of Family Medicine, Queen’s University, Kingston, ON K7L 3N6, Canada; 4 Division of Palliative Medicine, Department of Medicine, Queen’s University, Kingston, ON K7L 3N6, Canada

**Keywords:** palliative care, advanced cancer, patient-reported outcomes, acute care, symptom complexity

## Abstract

Among adult advanced cancer patients already accessing palliative care, symptoms can contribute to unplanned acute care utilizations, which can disrupt care and worsen patient outcomes. We examined how a novel symptom complexity algorithm, using patients’ ratings of the nine Edmonton Symptom Assessment System—Revised (ESAS-r) symptoms to assign “low”, “medium”, or “high” complexity, predicts acute care utilizations. This retrospective observational cohort study used electronic medical record data from the Durham Regional Cancer Centre in Ontario, Canada, comprising adult advanced cancer patients who completed at least one ESAS-r report between 1 January 2022 and 31 December 2023. We applied chi-squared tests, Kruskal–Wallis H tests, and multivariable binary logistic regressions to evaluate factors associated with higher odds of acute care utilization within seven and fourteen days of patients’ first ESAS-r reports after their first palliative care interaction. Of 559 included patients, 125 (22.4%) exhibited low complexity, 180 (32.2%) exhibited medium complexity, and 254 (45.4%) exhibited high complexity on their first ESAS-r report. In total, 61 (10.9%) patients accessed acute care within seven days and 108 (19.3%) patients accessed acute care within fourteen days of their first ESAS-r report. Controlling for sociodemographic and clinical covariates, compared to low-complexity patients, high-complexity patients had higher odds of acute care utilization within seven days (aOR = 2.83, 95% CI: 1.18–6.77), but not within fourteen days (aOR = 1.78, 95% CI: 0.97–3.28). Accordingly, as a clinical decision-making tool, ESAS-r symptom complexity may help identify patients who would benefit from more intensive follow-up and potentially reduce unnecessary acute care utilizations.

## Simple Summary

Advanced cancer symptoms can cause patients to go to the emergency department or stay in the hospital. Clinicians can use the Edmonton Symptom Assessment System—Revised (ESAS-r) to monitor these symptoms. We investigated if a new “symptom complexity” algorithm, which uses the ESAS-r to identify patients with “low”, “medium”, or “high” symptom complexity, could predict whether adult advanced cancer patients will use these hospital services. Of 559 patients, we identified 125 (22.4%) low-complexity, 180 (32.2%) medium-complexity, and 254 (45.4%) high-complexity patients. In total, 61 (10.9%) patients used these hospital services in seven days and 108 (19.3%) used them in fourteen days. High-complexity patients were 2.83 times more likely than low-complexity patients to access these hospital services within seven days. However, these groups of patients used these services equally as often within fourteen days. Subsequently, this algorithm may help clinicians identify patients who need more frequent follow-ups to prevent unnecessary hospital visits.

## 1. Introduction

Palliative care (PC) is an interdisciplinary medical approach that improves the quality of life of patients and caregivers dealing with life-limiting illnesses and enhances community-based end-of-life care [1,2,3]. In practice, PC incorporates symptom management and psychosocial support to provide comprehensive treatment that focuses on relieving, rather than curing, symptoms and suffering [4,5]. Patients and caregivers who are dealing with advanced cancer, defined as metastatic stage 3 or 4 cancer that is unlikely to be cured, significantly benefit from such treatments and generate demand for high-quality PC delivered parallel to curative therapy throughout their clinical trajectory to improve the client experience and quality of life [2,6,7,8]. Since 2007, Ontario Health has systematically assessed patients living with cancer using the patient-reported Edmonton Symptom Assessment System tools in Ontario’s fourteen regionalized cancer centres. The original Edmonton Symptoms Assessment System was developed in 1991 by Bruera et al. It uses visual analog scales to individually quantify patient burden for eight mandatory advanced cancer symptoms, namely pain, tiredness, nausea, depression, anxiety, drowsiness, appetite, and dyspnea [9,10,11,12,13,14]. In 2015, the Edmonton Symptom Assessment System—Revised (ESAS-r) was updated to quantify burden using an eleven-point Likert scale and include a ninth mandatory symptom, well-being [15].

**Figure 1:**
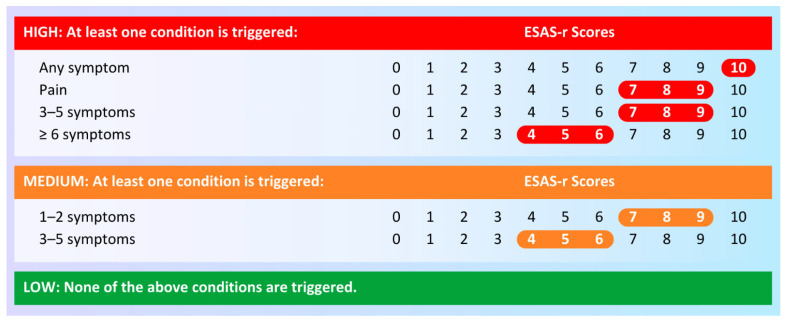
Visualization of the ESAS-r symptom complexity algorithm [18] Patients have high symptom complexity if at least one of four conditions are triggered: any symptom is rated 10 out of 10, pain is rated between 7 and 9 out of 10, three to five symptoms are rated between 7 and 9 out of 10, or six or more symptoms are rated between 4 and 6 out of 10. Patients have medium symptom complexity if no high symptom complexity conditions are triggered and at least one of two conditions are triggered: one to two symptoms are rated between 7 and 9 out of 10 or three to five symptoms are rated between 4 and 6 out of 10. Patients have low symptom complexity if none of these six conditions are triggered.

Previous literature supports the efficacy of individual patient-reported symptom screening scores in predicting care outcomes, especially acute care utilizations. Notably, a 2021 retrospective study by Noel et al. on adult head and neck cancer patients in the Ontario Cancer Registry concluded that specific ESAS-r symptom scores are strongly positively associated with emergency department uses and hospital admissions within fourteen days of outpatient ESAS-r administration [16]. More recent literature indicates the possibility of implementing ESAS-r symptom complexity, a proposed, more clinician-friendly screening methodology. In 2020, Watson et al. proposed a new symptom complexity algorithm that considers the presence, number, and severity of the individual ESAS-r symptom scores to stratify patients as having “low”, “medium”, or “high” complexity (Figure 1) [17]. Their 2023 cohort study of multi-stage adult cancer patients in Alberta, Canada demonstrated that this proposed three-level ordinal variable was positively associated with emergency department and hospital admissions within 7 days of ESAS-r administration [18]. However, it is not clear whether this relationship holds for patients who are already accessing outpatient PC services. Therefore, our study seeks to examine how ESAS-r symptom complexity predicts acute care utilizations among adult advanced cancer patients who are already accessing PC services in a community setting.

## 2. Materials and Methods

### 2.1. Study Context

At the Durham Regional Cancer Centre (DRCC), adult advanced cancer patients receive a referral to the PC clinic if they fulfill any of these criteria: (1) the patient has active symptoms requiring ongoing monitoring, (2) the patient has reviewed their palliative or end-of-life care needs, (3) the patient has participated in advanced care planning or goals of care discussions, or (4) if the patient does not have any active symptoms or other care needs, the patient has metastatic cancer with a prognosis less than six months.

Cancer patients can complete the ESAS-r before their appointment by independently completing a digitized assessment on patient kiosks in clinic waiting rooms or verbally completing the assessment with the assistance of a clinician who will transcribe scores onto the assessment form [9,19]. Subsequently, in a patient’s electronic medical record, the completed ESAS-r report is shown alongside a summary of available previous ESAS-r assessments for review by their oncology clinicians [9].

### 2.2. Study Design and Population

The current study uses a retrospective observational cohort to analyze associations between ESAS-r symptom complexity and acute care utilization. The inclusion criteria comprised adult DRCC patients with an advanced cancer diagnosis who accessed PC services within the DRCC and completed at least one ESAS-r report between 1 January 2022 and 31 December 2023. Advanced cancer diagnoses were identified based on oncologist documentation with mention of the terms ‘advanced’ or ‘metastatic.’ Subsequently, we selected the patient’s first ESAS-r report after their first interaction with a PC service or flag on the electronic medical record.

### 2.3. Exposure and Outcomes

The primary exposure was the ordinal ESAS-r symptom complexity score, calculated from the symptom scores in the patient’s first ESAS-r report. The two primary outcomes were acute care utilizations (ACUs), defined as emergency department (ED) uses or hospital admissions within seven days and fourteen days after their first ESAS-r report, both codified as binary variables. A secondary outcome assessed the length of survival stratified by the primary exposure.

### 2.4. Covariates

Regarding sociodemographic characteristics, age, sex, income, primary language for clinical care, and rurality were included. Income was derived from the median income of patients’ local region of residence and sorted into equal-size quartiles (Q1: 29,200–36,400 CAD, Q2: 36,400–42,800 CAD, Q3: 42,800–48,400 CAD, Q4: 48,400–66,000 CAD) and rurality was collapsed into urban and rural residences. The Ontario Marginalization Index (ON-Marg) summary score and an adjusted Charlson comorbidity index (CCI) score that excluded age- and cancer-related comorbidities were also examined [20]. The ON-Marg summary score is assigned by the Government of Ontario to adequately populous postal code regions to quantify residential instability, material deprivation, dependency, and ethnic concentration, and is widely used for administrative database and epidemiologic studies in the province with supported validity as a comprehensive tool to study health inequities in Ontario [21], albeit with limitations due to variability in marginalization between residents of the same postal code [22].

Regarding clinical characteristics, the latest tumour location, the use of chemotherapy and radiotherapy in the 30 days preceding the ESAS-r report, the location of the patient’s first PC visit, the involvement of home care, the use of the PC unit, and survival and location of death until the end of the observation period were reviewed as categorical variables. Additionally, the number of days from the latest cancer diagnosis to the first PC interaction, from the latest diagnosis to the first ESAS-r report, from the first PC interaction to the first ESAS-r report, and from the first ESAS-r report to death (survival) were calculated as continuous variables.

### 2.5. Statistical Analysis

Bivariate descriptive statistics were applied to summarize patient characteristics. Statistical significance was investigated using Pearson chi-squared tests for categorical variables and Kruskal–Wallis H tests for continuous variables. Multivariable logistic regression models were created for each primary outcome to produce adjusted odds ratios (aOR) to control specific covariates.

Kaplan–Meier survival curves were produced for the secondary outcome. Patients experienced an event if they passed away during a six-month post-observation period ending on 30 June 2024 or were censored otherwise.

Microsoft Excel version 16.86 and SPSS Statistics version 29.0.2 (IBM Corp., Armonk, USA) were used for statistical analysis. Statistical significance was set *a priori* at *p* < 0.05.

### 2.6. Ethics

Ethics approval was obtained from the Queen’s University Health Sciences and Affiliated Teaching Hospitals Ethics Board (#6028581) and Lakeridge Health Research Ethics Board (#2019-011).

## 3. Results

### 3.1. Symptom Complexity

A total of 559 patients met our inclusion criteria (Table 1). Of these, 125 (22.4%) patients exhibited low ESAS-r symptom complexity (low-complexity), 180 (33.2%) patients exhibited medium ESAS-r symptom complexity (medium-complexity), and 254 (45.4%) patients exhibited high ESAS-r symptom complexity (high-complexity) on their first ESAS-r report after their first interaction with PC. Stratifying the cohort by symptom complexity revealed some statistically significant differences in patients’ sociodemographic and clinical characteristics (Table S1), with high-complexity patients speaking English more frequently (46.3%) and having used chemotherapy most in the thirty days preceding their ESAS-r report (38.0%). No significant differences were identified regarding age, sex, tumour site, the involvement of home care, the use of the PC unit, or the location and timing of death, though it is noted that high-complexity patients comprised most patients who passed away between 0–30 days (53.9%) and 31–90 days (53.0%) and a plurality of those who passed beyond 91 days (41.6%).

### 3.2. Acute Care Utilization Within 7 Days

Only 61 (10.9%) patients had an ACU within 7 days of their first ESAS-r report, of which 14 (23.0%) accessed the PC unit. Bivariate analysis (Table 2; Table S2) demonstrated that patients who accessed acute care had higher symptom complexity, with seventeen (9.4%) medium-complexity patients and thirty-seven (14.6%) high-complexity patients accessing acute care compared to seven (5.6%) low-complexity patients. Additionally, patients who accessed acute care used chemotherapy less in the thirty days before their ESAS-r report (18.0%), had their first interaction with PC sooner (median 21 days), and completed their first ESAS-r report sooner (median 20 days) after their latest tumour diagnosis. Finally, patients with an ACU within 7 days passed away sooner after their first ESAS-r report (median 25 days) with 47.5% dying within 30 days, and more often in hospital (70.5%) relative to patients without an ACU (34.5%). Only 23.0% lived longer than 90 days versus 36.7% of patients without an ACU. Furthermore, most patients who accessed PC services (89.1%) or home-based PC (88.6%) did not incur an ACU within 7 days.

Multivariable analysis (Table 3; Table S3) revealed that only high-complexity patients had significantly greater odds of accessing acute care (aOR = 2.83, 95% CI: 1.18–6.77). Patients with an ACU within 7 days also had their first PC interaction sooner after their latest tumour diagnosis (aOR = 1.00, 95% CI: 1.00–1.00) and used less chemotherapy (aOR = 0.48, 95% CI: 0.23–0.98).

### 3.3. Acute Care Utilization Within 14 Days

A total of 108 (19.3%) patients had an ACU within 14 days of their first ESAS-r report, indicating that an additional 47 (8.4%) patients accessed either service during the second week following their report. Twenty-five (23.1%) of these patients accessed the PC unit. Compared to the seven-day bivariate analysis, the fourteen-day bivariate analysis (Table 4; Table S4) maintained significant differences in symptom complexity, with 28 (15.6%) medium-complexity patients and 62 (24.4%) high-complexity patients accessing acute care compared to 18 (14.4%) low-complexity patients. Moreover, similarly, patients with ACUs had a first interaction with PC sooner (median 35 days) and completed their first ESAS-r report sooner after their latest tumour diagnosis (median 34 days). These patients also passed away at a higher rate between 0 and 30 days (39.8%). However, these patients also accessed more radiotherapy in the thirty days preceding their ESAS-r assessment (21.3%). Finally, most patients who accessed PC services (80.7%) or home-based PC (79.5%) did not incur an ACU within 14 days.

Multivariable analysis (Table 5; Table S5) did not reveal significant differences in the odds of ACU among symptom complexity levels. Instead, patients with advanced gynaecological cancer were 4.55 times (95% CI: 1.21–17.04) more likely than advanced breast cancer patients to access acute care. Additionally, similar to the seven-day multivariable analysis, patients also had their first PC interaction sooner after their latest tumour diagnosis (aOR = 1.00, 95% CI: 1.00–1.00).

### 3.4. Survival over 6 Months

**Figure 2:**
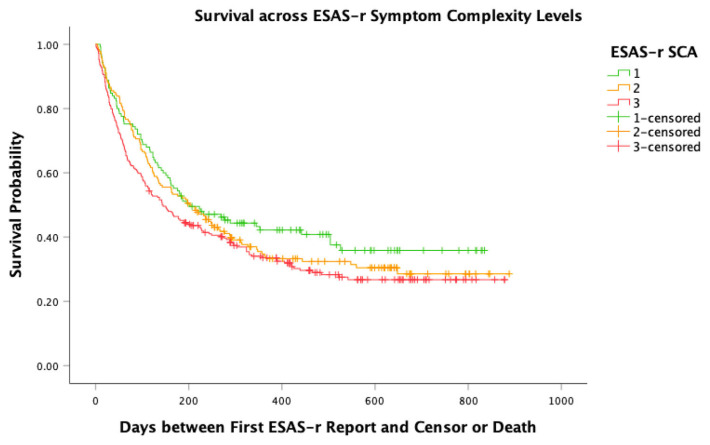
Kaplan–Meier curves of survival across ESAS-r symptom complexity levels.

The Kaplan–Meier bivariate log-rank test did not indicate significant differences between the survival distributions across the low-complexity, medium-complexity, and high-complexity patients (χ^2^ = 4.19, *p* = 0.12). In total, 40.0% of low-complexity patients, 33.3% of medium-complexity patients, and 30.7% of high-complexity patients survived until the end of the observation period (Figure 2).

## 4. Discussion

The current study demonstrates that high ESAS-r symptom complexity, derived using an algorithm applied to the first set of individual ESAS-r symptom scores for patients that have already accessed PC, is associated with an increased likelihood of ACUs within seven days of the ESAS-r report, but not within fourteen days. These results indicate an association similar to that found in a 2023 province-wide study with a larger, multi-stage cancer cohort [18].

The association over the seven-day period suggests that even when PC is involved, adult advanced cancer patients with high symptom complexity still access more acute care services. This differential use might indicate more need for acute care services despite community PC team involvement. There might also be difficulty accessing timely and adequate symptom management through their PC physician, oncologist, family physician, or other healthcare providers [23]. A provincial study in Alberta revealed that most ED visits among adult PC patients were made by patients with self-reported unmet PC needs [24]. These patients subsequently underwent more consultations, hospital admissions, and deaths in hospital settings, and received fewer referrals to PC during and after their ED visits [24].

High symptom complexity also indicates poorer prognoses. This advanced cancer population seeing PC services also had a larger proportion (45.4%) of patients with high symptom complexity, compared to only 13.7% in Watson et al.’s all-stage cancer cohort [18]. It is known that symptoms get worse as cancer progresses; thus, in conjunction with increased ACUs, poor prognosis may reflect difficulties in managing severe advanced cancer symptoms and complications at home and integrating lay and home-based care methods and signal the necessity of increased clinician support toward the end of life [2,5]. Moreover, as evidenced by the higher rate of admissions and death in the hospital PC unit, a substantial number of patients who access PC may still require acute care visits [25]. Subsequently, ESAS-r symptom complexity could inform how PC and other symptom management and psychosocial support services should be allocated, including the types, frequencies, and delivery methods of these services.

Most patients who accessed PC services, including home-based PC, did not access acute care in either seven days or fourteen days. These results align with retrospective provincial studies conducted among Ontario adult PC patients which indicate that integrating home care into a PC regimen produces significant reductions in a patient’s subsequent need to access acute care [25,26]. In particular, these studies suggest that home visits facilitate early PC intervention with better management of complex symptoms and prevention of high-acuity issues [27]. Such visits may also empower conversations about goals of care, help patients and caregivers prepare for the dying process, and motivate patients to opt for home care over hospital-based acute care [25,26,27,28,29].

While physician-referred medically necessary PC services are insured under the Ontario Health Insurance Plan and family-based health promotion interventions like advanced cancer planning and the Compassionate Care Act are helping patients access PC earlier [30], Ontario patients with advanced cancer still experience delays in accessing PC services. Subsequently, clinician-administered, patient-reported PC needs assessments may be vital in informing service allocation [2,4]. Accordingly, the current ESAS-r symptom complexity algorithm may provide a standardized referral or triage methodology for PC services that optimizes the allocation of Ontario’s PC clinicians [9,10,17,31]. For instance, lower-complexity patients could receive early PC through primary healthcare providers, while higher-complexity patients could receive more intense PC through oncologists and PC physicians [8]. For those on the PC teams, more frequent follow-ups can be prioritised for those with higher symptom complexity. The necessity of triage is amplified by the increasing prevalence of cancer (5478.9 per 100,000 persons in 2018 to 5612.5 per 100,000 persons in 2034, constituting a 2.44% increase over 16 years) [1]. Additionally, advanced cancer patients are also living longer with distressing symptoms, reinforcing the importance of prioritizing limited PC clinician resources [2,8,11,28,32,33].

In clinical practice, an ESAS-r symptom complexity-based triage system could work alongside other clinical informatics tools to facilitate clinical decision-making [17,18,34]. Downstream, it may promote interprofessional collaboration and proactive care and maximize patients’ physical functioning, emotional health, and quality of life [2,8,9,12,27,31,33,35,36]. The addition of a complexity tool may simplify clinical decision-making for busy clinicians. Earlier PC follow-up would facilitate symptom-focused discussions between clinicians and patients and allow intensified PC interventions within the seven-day window [37]. These interventions could include increased frequency of symptom screening, early and increased home care, early prescriptions for palliative symptom relief kits for patients with high symptom complexity and poor prognosis, and psychosocial support for caregivers.

The results of the current study must be interpreted in light of limitations and approximations made. First, information on potentially important covariates such as proxy completion of the ESAS-r tool, one-person household status, immigration status, smoking status, and functional status could not be collected. However, the ON-Marg summary score and the adjusted CCI were included to represent some of these variables. While the ON-Marg summary score is not necessarily accurate to the individual, it is commonly used in epidemiologic studies in the province of Ontario and is sufficiently valid to apply toward studying health inequalities [21]. Second, we did not determine whether a patient possessed a family physician or nurse practitioner, or whether an oncologist was still involved in a patient’s care prior to their first PC interaction at the DRCC. These clinicians may also be providing PC. However, home care involvement was included as a covariate. Third, the nature, acuity, or relationship of ED visits and non-PC unit admissions to advanced cancer and palliative symptoms could not be verified. Finally, it is important to note that symptom complexity does not necessary correlate with ‘medical complexity’ or ‘care complexity’; these holistic forms of complexity would incorporate additional dimensions of complexity, including but not limited to comorbidities, functional impairments, social situation, socioeconomic disparity, and treatment responsiveness.

## 5. Conclusions

Among adult advanced cancer patients who access outpatient PC services, ESAS-r symptom complexity predicts higher ACUs for the first week following the first ESAS-r report but not the first two weeks. Subsequently, the determination and assignment of symptom complexity ratings to patients upon completing the ESAS-r may serve as an additional tool for clinicians to determine optimal PC symptom management and follow-up plans, with an emphasis on timeliness, intensiveness, comprehensiveness, and responsiveness to patient-reported outcomes. Future studies should investigate trends in symptom complexity over time or how the intensiveness of PC follow-up might impact acute care utilization.

## Figures and Tables

**Table 1:** Patient characteristics.

Characteristic *n* = 559	Whole Cohort *n* (Col %)
Total	559 (100.0)
Age, mean [SD], years	71 [13]
Sex	
Female	284 (50.8)
Male	275 (49.2)
Language	
English	547 (97.9)
Other	12 (2.1)
Tumour site	
Breast	50 (8.9)
Gastrointestinal	130 (23.3)
Genitourinary	62 (11.1)
Gynaecological	34 (6.1)
Haematological	36 (6.4)
Lung	92 (16.5)
Other *	155 (27.7)
Used chemotherapy in 30 days prior to ESAS-r?	166 (29.7)
Location of first PC visit	
Clinic	526 (94.1)
Other **	33 (5.9)
Used home care?	132 (23.6)
Latest diagnosis to first PC interaction, median [IQR], days (n = 538, 96.2%)	74 [194]
Latest diagnosis to first ESAS-r report, median [IQR], days (n = 538, 96.2%)	77 [189]
Location of death	
Hospital †	215 (38.5)
Non-hospital	171 (30.6)
Alive or missing ‡	173 (30.9)
Survival, median [IQR], days (n = 386, 69.1%)	97 [160]
Survival	
Survived 0–30 days	89 (15.9)
Survived 31–90 days	100 (17.9)
Survived beyond 90 days	197 (35.2)
Alive or missing ‡	173 (30.9)

* Includes central nervous system, endocrine, head and neck, bone and soft tissue (sarcoma), skin, and unknown tumours; ** includes inpatient hospital, home visit, and no first PC visit setting; † note that patients did not necessarily die during their initial hospitalization; ‡ either did not die during observation period, or death not captured in database.

**Table 2:** Patient characteristics stratified by acute care utilizations within 7 days.

Characteristic (*n* = 559)	Emergency Department Visit or Hospital Admission Within 7 Days		
	No n (Col %)	Yes n (Col %)	*p* Value
Total (Row %)	498 (89.1)	61 (10.9)	
PC unit	-	14 (23.0)	<0.01
Other unit	-	47 (77.0)	
Symptom complexity (Row %)			
Low	**118 (94.4)**	**7 (5.6)**	**0.02**
Medium	**163 (90.6)**	**17 (9.4)**	
High	**217 (85.4)**	**37 (14.6)**	
Age, mean [SD], years	71 [13]	70 [13]	0.64
Sex			
Female	254 (51.0)	30 (49.2)	0.79
Male	244 (49.0)	31 (50.8)	
Language			
English	486 (97.6)	61 (100.0)	0.22
Other	12 (2.4)	0 (0.0)	
Tumour site			
Breast	46–50	1–5	0.75
Gastrointestinal	115 (23.1)	15 (24.6)	
Genitourinary	56 (11.2)	6 (9.8)	
Gynaecological	26–30	1–5	
Haematological	31–35	1–5	
Lung	78 (15.7)	14 (23.0)	
Other *	139 (27.9)	16 (26.2)	
Used chemotherapy in 30 days prior to ESAS-r?	**155 (31.1)**	**11 (18.0)**	**0.04**
Location of first PC visit			
Clinic	468 (94.0)	58 (95.1)	0.73
Other **	26–30	1–5	
Used home care?	117 (23.5)	15 (24.6)	0.85
Latest diagnosis to first PC interaction, median [IQR], days	**81** [196] **(n = 479)**	**21** [80] **(n = 59)**	**<0.01**
Latest diagnosis to first ESAS-r report, median [IQR], days	**85** [192] **(n = 479)**	**20** [101] **(n = 59)**	**<0.01**
Location of death			
Hospital	**172 (34.5)**	**43 (70.5)**	**<0.01**
Non-hospital	**164 (32.9)**	**7 (11.5)**	
Alive or missing ‡	**162 (32.5)**	**11 (18.0)**	
Survival, median [IQR], days	**101** [173] **(n = 336, 67.5%)**	**25** [96] **(n = 50, 82.0%)**	**<0.01**
Survival †			
Survived 0–30 days	**60 (12.0)**	**29 (47.5)**	**<0.01**
Survived 31–90 days	**93 (18.7)**	**7 (11.5)**	
Survived beyond 90 days	**183 (36.7)**	**14 (23.0)**	
Alive or missing ‡	**162 (32.5)**	**11 (18.0)**	

* Includes central nervous system, endocrine, head and neck, bone and soft tissue (sarcoma), skin, and unknown tumours; ** includes inpatient hospital, home visit, and no first PC visit setting; † note that patients did not necessarily die during their hospitalization; ‡ either did not die during observation period, or death not captured in database.

**Table 3:** Multivariable binary logistic regression of ESAS-r symptom complexity and acute care utilization within 7 days.

Characteristic (*n* = 559)	Emergency Department Visit or Hospital Admission Within 7 Days	
	aOR (95% CI)	*p* Value
Symptom complexity		
Low	Ref	
Medium	1.56 (0.60–4.05)	0.37
High	**2.83 (1.18–6.77)**	**0.02**
Sex		
Female	Ref	
Male	1.18 (0.62–2.23)	0.62
Tumour site		
Breast	Ref	
Gastrointestinal	1.83 (0.45–7.43)	0.40
Genitourinary	1.51 (0.31–7.33)	0.61
Gynaecological	2.63 (0.51–13.45)	0.25
Haematological	1.32 (0.22–7.77)	0.76
Lung	2.69 (0.66–10.98)	0.17
Other ***	1.78 (0.45–7.07)	0.42
Chemotherapy in 30 days prior to ESAS-r		
No	Ref	
Yes	**0.48 (0.23–0.98)**	**0.04**
Involvement of home care		
No	Ref	
Yes	0.89 (0.46–1.77)	0.76
Days from latest diagnosis to first PC interaction, median [IQR], days	**1.00 (1.00–1.00)**	**0.05**

*** includes central nervous system, endocrine, head and neck, bone and soft tissue (sarcoma), skin, and unknown tumours.

**Table 4:** Patient characteristics stratified by acute care utilizations within 14 days.

Characteristic (*n* = 559)	Emergency Department Visit or Hospital Admission Within 14 Days		
	No n (Col %)	Yes n (Col %)	*p* Value
**Total (Row %)**	451 (80.7)	108 (19.3)	
PC unit	-	25 (23.1)	< 0.01
Other unit	-	83 (76.9)	
Symptom complexity (Row %)			
Low	**107 (85.6)**	**18 (14.** **4)**	**0.02**
Medium	**152 (84.4)**	**28 (15.6)**	
High	**192 (75.6)**	**62 (24.4)**	
Age, mean [SD], years	71 [13]	70 [12]	0.33
Sex			
Female	237 (52.5)	47 (43.5)	0.09
Male	214 (47.5)	61 (56.5)	
Language			
English	440 (97.6)	107 (99.1)	0.33
Other	11–15	1–5	
Tumour site			
Breast	46–50	1–5	0.33
Gastrointestinal	108 (23.9)	22 (20.4)	
Genitourinary	47 (10.4)	15 (13.9)	
Gynaecological	25 (5.5)	9 (8.3)	
Haematological	29 (6.4)	7 (6.5)	
Lung	73 (16.2)	19 (17.6)	
Other *	123 (27.3)	32 (29.6)	
Used radiotherapy in 30 days prior to ESAS-r?	**58 (12.9)**	**23 (21.3)**	**0.03**
Location of first PC visit			
Clinic	424 (94.0)	102 (94.4)	0.86
Other **	27 (6.0)	6 (5.6)	
Used home care?	105 (23.3)	27 (25.0)	0.71
Days from diagnosis to first PC interaction, median [IQR], days	**86** [197] **(n = 432)**	**35** [99] **(n = 106)**	**<0.01**
Days from diagnosis to first ESAS-r report, median [IQR], days	**88** [202] **(n = 432)**	**34** [105] **(n = 106)**	**<0.01**
Location of death			
Hospital	**149 (33.0)**	**66 (61.1)**	**<** **0.01**
Non-hospital	**146 (32.4)**	**25 (23.1)**	
Alive or missing ‡	**156 (34.6)**	**17 (15.7)**	
Survival, median [IQR], days	**107** [177] **(n = 295)**	**36** [100] **(n = 91)**	**<0.01**
Survival †			
0–30	**46 (10.2)**	**43 (39.8)**	**<0.01**
31–90	**83 (18.4)**	**17 (15.7)**	
91+	**166 (36.8)**	**31 (28.7)**	
Alive or missing ‡	**156 (34.6)**	**17 (15.7)**	

* Includes central nervous system, endocrine, head and neck, bone and soft tissue (sarcoma), skin, and unknown tumours; ** includes inpatient hospital, home visit, and no first PC visit setting; † note that patients did not necessarily die during their hospitalization; ‡ either did not die during observation period, or death not captured in database.

**Table 5:** Multivariable binary logistic regression of ESAS-r symptom complexity and acute care utilization within 14 days.

Characteristic (*n* = 559)	Emergency Department Visit or Hospital Admission Within 14 Days	
	aOR (95% CI)	*p* Value
Symptom complexity		
Low	Ref	
Medium	0.99 (0.50–1.95)	0.98
High	1.78 (0.97–3.28)	0.07
Sex		
Female	Ref	
Male	1.54 (0.92–2.58)	0.10
Tumour site		
Breast	Ref	
Gastrointestinal	1.59 (0.48–5.30)	0.45
Genitourinary	2.49 (0.67–8.88)	0.17
Gynaecological	**4.55 (1.21–17.04)**	**0.03**
Haematological	2.29 (0.56–9.32)	0.25
Lung	2.11 (0.62–7.14)	0.23
Other *	2.57 (0.80–8.24)	0.11
Radiotherapy in 30 days prior to ESAS-r		
No	Ref	
Yes	1.70 (0.95–3.04)	0.07
Involvement of home care		
No	Ref	
Yes	1.02 (0.60–1.72)	0.96
Days from latest diagnosis to first PC interaction, median [IQR], days	**1.00 (1.00–1.00)**	**<0.01**

* Includes central nervous system, endocrine, head and neck, bone and soft tissue (sarcoma), skin, and unknown tumours.

## Data Availability

The data presented in this study are available on request from the corresponding author. The data set from this study is held securely in coded at Lakeridge Health. While the conditions of our ethics approval prohibit making the data set publicly available, access to anonymized summary-level aggregate data may be granted, conditional on permission from the data custodian, upon request by emailing asinnarajah@lh.ca, understanding that the programs may rely on coding templates or macros that are unique to Lakeridge Health and this study.

## Supplementary Materials

The following supporting information can be downloaded at https://www.mdpi.com/article/10.3390/curroncol32070388/s1, Table S1: Patient characteristics stratified by ESAS-r symptom complexity level; Table S2: Patient characteristics stratified by acute care utilizations within 7 days; Table S3: Multivariable binary logistic regression of ESAS-r symptom complexity and acute care utilization within 7 days; Table S4: Patient characteristics stratified by acute care utilizations within 14 days; Table S5: Multivariable binary logistic regression of ESAS-r symptom complexity and acute care utilization within 14 days.
