# Supplementary material for: Associations Between Symptom Complexity and Acute Care Utilization Among Adult Advanced Cancer Patients Followed by a Palliative Care Service
Source: Curr Oncol. 2025 Jul 4;32(7):388. doi: 10.3390/curroncol32070388 (PMC12293374; doi:10.3390/curroncol32070388)
Supplement: Supplementary file 1 [file curroncol-32-00388-s001.zip › curroncol-3714118-supplementary.pdf]

Current Oncology

MDPI

Supplementary Materials:

Associations Between Symptom Complexity and Acute Care Utilization Among Advanced Cancer Patients Followed by a Palliative Care Service

Philip Pranajaya [1], Vincent Ho [2,3], Mengzhu Jiang [2,3], Vance Tran [2,3] and Aynharan Sinnarajah [2,4,*]

[1] Faculty of Health Sciences, Queen's University, Kingston, ON, Canada
[2] Division of Palliative Medicine, Department of Medicine, Lakeridge Health, Oshawa, ON, Canada
[3] Department of Family Medicine, Queen's University, Kingston, ON, Canada
[3] Division of Palliative Medicine, Department of Medicine, Queen's University, Kingston, ON, Canada
* Correspondence: asinnarajah@lh.ca

Table S1. Patient characteristics stratified by ESAS-r symptom complexity level.

Characteristic n = 559	Whole cohort n (Col %)	Symptom complexity level			p value
		Low n (Row %)	Medium n (Row %)	High n (Row %)	
**Total**	**559 (100.0)**	125 (22.4)	180 (32.2)	254 (45.4)	
Age, mean [SD], y	71 [13]	69 [12]	72 [13]	71 [12]	0.06
Age categories					
20-55	52 (9.3)	13 (25.0)	14 (26.9)	25 (48.1)	0.24
56-65	151 (27.0)	45 (29.8)	44 (29.1)	62 (41.1)	
66-75	134 (24.0)	25 (18.7)	41 (30.6)	68 (50.7)	
76-85	149 (26.7)	30 (20.1)	55 (36.9)	64 (43.0)	
86+	73 (13.1)	12 (16.4)	26 (35.6)	35 (47.9)	
Sex					
Female	284 (50.8)	63 (22.2)	86 (30.3)	135 (47.5)	0.54
Male	275 (49.2)	62 (22.5)	94 (34.2)	119 (43.3)	
Income quartile*					
Q1	140 (25.0)	25 (17.9)	39 (27.9)	76 (54.3)	0.25
Q2	140 (25.0)	31 (22.1)	48 (34.3)	61 (43.6)	
Q3	140 (25.0)	38 (27.1)	48 (34.3)	54 (38.6)	
Q4	139 (24.9)	31 (22.3)	45 (32.4)	63 (45.3)	
Language					
English	547 (97.9)	**123 (22.5)**	**171 (31.3)**	**253 (46.3)**	**< 0.01**
Other	12 (2.1)	**1–5**	**6–10**	**1–5**	
Rurality					
Rural	15 (2.7)	1–5	1–5	6–10	0.49
Urban	544 (97.3)	123 (22.6)	176 (32.4)	245 (45.0)	
Ontario Marginalization (ON-Marg) Index summary score (n = 542)					
1-2	85 (15.2)	27 (31.8)	25 (29.4)	33 (38.8)	0.45
3	223 (39.9)	43 (19.3)	75 (33.6)	105 (47.1)	
4	198 (35.4)	43 (21.7)	61 (30.8)	94 (47.5)	
5	36 (6.4)	9 (25.0)	11 (30.6)	16 (44.4)	

Missing**	17 (3.0)	1–5	6–10	6–10	
Adjusted Charlson Comorbidity Index (CCI) score					
0	456 (81.6)	106 (23.2)	143 (31.4)	207 (45.4)	0.49
1+	103 (18.4)	19 (18.4)	37 (35.9)	47 (45.6)	
Tumour site					
Breast	50 (8.9)	14 (28.0)	14 (28.0)	22 (44.0)	0.07
Gastrointestinal	130 (23.3)	37 (28.5)	48 (36.9)	45 (34.6)	
Genitourinary	62 (11.1)	12 (19.4)	13 (21.0)	37 (59.7)	
Gynaecological	34 (6.1)	10 (29.4)	8 (23.5)	16 (47.1)	
Haematological	36 (6.4)	1–5	16–20	11–15	
Lung	92 (16.5)	15 (16.3)	31 (33.7)	46 (50.0)	
Other***	155 (27.7)	32 (20.6)	50 (32.3)	73 (47.1)	
Used chemotherapy in 30 days prior to ESAS-r?	166 (29.7)	**50 (30.1)**	**53 (31.9)**	**63 (38.0)**	**0.01**
Used radiotherapy in 30 days prior to ESAS-r?	81 (14.5)	15 (18.5)	23 (28.4)	43 (53.1)	0.32
Location of first PC visit					
Clinic	526 (94.1)	115 (21.9)	169 (32.1)	242 (46.0)	0.44
Other****	33 (5.9)	10 (30.3)	11 (33.3)	12 (36.4)	
Used home care?	132 (23.6)	30 (22.7)	43 (32.6)	59 (44.7)	0.98
Used PC unit within 7 days of first ESAS-r report?	14 (2.5)	1–5	1–5	6–10	0.37
Used PC unit within 14 days of first ESAS-r report?	25 (4.5)	1–5	6–10	16–20	0.10
Latest diagnosis to first PC interaction, median [IQR], days (n = 538)	74 [194]	77 [239] (n = 123)	79 [181] (n = 173)	69 [185] (n = 242)	0.38
Latest diagnosis to first ESAS-r report, median [IQR], days (n = 538)	77 [189]	87 [234] (n = 123)	79 [181] (n = 173)	69 [170] (n = 242)	0.25
Location of death					
Hospital†	215 (38.5)	45 (20.9)	69 (32.1)	101 (47.0)	0.54
Non-hospital	171 (30.6)	34 (19.9)	55 (32.2)	82 (48.0)	
Alive or missing‡	173 (30.9)	46 (26.6)	56 (32.4)	71 (41.0)	
Survival, median [IQR], days (n = 386, 69.1%)	97 [160]	109 [149] (n = 79, 63.2%)	108 [166] (n = 124, 68.9%)	69 [149] (n = 183, 72.0%)	0.15
Survival					
Survived 0-30 days	89 (15.9)	17 (19.1)	24 (27.0)	48 (53.9)	0.19
Survived 31-90 days	100 (17.9)	18 (18.0)	29 (29.0)	53 (53.0)	
Survived beyond 90 days	197 (35.2)	44 (22.3)	71 (36.0)	82 (41.6)	
Alive or missing‡	173 (30.9)	46 (26.6)	56 (32.4)	71 (41.0)	

* Q1: 29200–36400 CAD, Q2: 36400–42800 CAD, Q3: 42800–48400 CAD, Q4: 48400–66000 CAD ; ** Includes patients residing in postal codes too small to be assigned a marginalization score; *** Includes central nervous system, endocrine, head and neck, bone and soft tissue (sarcoma), skin, and unknown tumours; **** Includes inpatient hospital, home visit, and no first PC visit setting; † Note that patients did not necessarily die during their initial hospitalization; ‡ Either did not die during observation period, or death not captured in database.

Table S2. Patient characteristics stratified by acute care utilizations within 7 days.

**Characteristic (n = 559)**	**Emergency department visit or hospital admission within 7 days**		
	**No n (Col %)**	**Yes n (Col %)**	**p value**
**Total (Row %)**	498 (89.1)	61 (10.9)	
PC unit	-	14 (23.0)	< 0.01
Other unit	-	47 (77.0)	
Symptom complexity (Row %)			
Low	**118 (94.4)**	**7 (5.6)**	**0.02**
Medium	**163 (90.6)**	**17 (9.4)**	
High	**217 (85.4)**	**37 (14.6)**	
Age, mean [SD], y	71 [13]	70 [13]	0.64
Age categories			
20-55	47 (9.4)	5 (8.2)	0.83
56-65	131 (26.3)	20 (32.8)	
66-75	122 (24.5)	12 (19.7)	
76-85	133 (26.7)	16 (26.2)	
86+	65 (13.1)	8 (13.1)	
Sex			
Female	254 (51.0)	30 (49.2)	0.79
Male	244 (49.0)	31 (50.8)	
Income quartile*			
Q1	124 (24.9)	16 (26.2)	0.37
Q2	130 (26.1)	10 (16.4)	
Q3	121 (24.3)	19 (31.1)	
Q4	123 (24.7)	16 (26.2)	
Language			
English	486 (97.6)	61 (100.0)	0.22
Other	12 (2.4)	0 (0.0)	
Rurality			
Rural	11–15	1–5	0.76
Urban	485 (97.4)	59 (96.7)	
Ontario Marginalization (ON-Marg) Index summary score			
1-2	74 (14.9)	11 (18.0)	0.67
3	195 (39.2)	28 (45.9)	
4	181 (36.3)	17 (27.9)	
5	31–35	1–5	
Missing**	11–15	1–5	
Adjusted Charlson Comorbidity Index (CCI) score			
0	409 (82.1)	47 (77.0)	0.33
1+	89 (17.9)	14 (23.0)	
Tumour site			

Breast	46–50	1–5	0.75
Gastrointestinal	115 (23.1)	15 (24.6)	
Genitourinary	56 (11.2)	6 (9.8)	
Gynaecological	26–30	1–5	
Haematological	31–35	1–5	
Lung	78 (15.7)	14 (23.0)	
Other***	139 (27.9)	16 (26.2)	
Used chemotherapy in 30 days prior to ESAS-r?	**155 (31.1)**	**11 (18.0)**	**0.04**
Used radiotheapy in 30 days prior to ESAS-r?	69 (13.9)	12 (19.7)	0.22
Location of first PC visit			
Clinic	468 (94.0)	58 (95.1)	0.73
Other****	26–30	1–5	
Used home care?	117 (23.5)	15 (24.6)	0.85
Latest diagnosis to first PC interaction, median [IQR], days	**81 [196] (n = 479)**	**21 [80] (n = 59)**	**< 0.01**
Latest diagnosis to first ESAS-r report, median [IQR], days	**85 [192] (n = 479)**	**20 [101] (n = 59)**	**< 0.01**
Location of death			
Hospital	**172 (34.5)**	**43 (70.5)**	**< 0.01**
Non-hospital	**164 (32.9)**	**7 (11.5)**	
Alive or missing‡	**162 (32.5)**	**11 (18.0)**	
Survival, median [IQR], days	**101 [173] (n = 336, 67.5%)**	**25 [96] (n = 50, 82.0%)**	**< 0.01**
Survival†			
Survived 0-30 days	**60 (12.0)**	**29 (47.5)**	**< 0.01**
Survived 31-90 days	**93 (18.7)**	**7 (11.5)**	
Survived beyond 90 days	**183 (36.7)**	**14 (23.0)**	
Alive or missing‡	**162 (32.5)**	**11 (18.0)**	

* Q1: 29200–36400 CAD, Q2: 36400–42800 CAD, Q3: 42800–48400 CAD, Q4: 48400–66000 CAD ; ** Includes patients residing in postal codes too small to be assigned a marginalization score; *** Includes central nervous system, endocrine, head and neck, bone and soft tissue (sarcoma), skin, and unknown tumours; **** Includes inpatient hospital, home visit, and no first PC visit setting; † Note that patients did not necessarily die during their hospitalization; ‡ Either did not die during observation period, or death not captured in database.

Table S3. Patient characteristics stratified by acute care utilizations within 14 days.

**Characteristic (n = 559)**	**Emergency department visit or hospital admission within 14 days**		
	**No n (Col %)**	**Yes n (Col %)**	**p value**
**Total (Row %)**	451 (80.7)	108 (19.3)	
PC unit	-	25 (23.1)	< 0.01
Other unit	-	83 (76.9)	
Symptom complexity (Row %)			

Low	**107 (85.6)**	**18 (14.4)**	**0.02**
Medium	**152 (84.4)**	**28 (15.6)**	
High	**192 (75.6)**	**62 (24.4)**	
Age, mean [SD], y	71 [13]	70 [12]	0.33
Age categories			
20-55	43 (9.5)	9 (8.3)	0.82
56-65	119 (26.4)	32 (29.6)	
66-75	106 (23.5)	28 (25.9)	
76-85	121 26.8)	28 (25.9)	
86+	62 (13.7)	11 (10.2)	
Sex			
Female	237 (52.5)	47 (43.5)	0.09
Male	214 (47.5)	61 (56.5)	
Income quartile*			
Q1	108 (23.9)	32 (29.6)	0.49
Q2	116 (25.7)	24 (22.2)	
Q3	111 (24.6)	29 (26.9)	
Q4	116 (25.7)	23 (21.3)	
Language			
English	440 (97.6)	107 (99.1)	0.33
Other	11–15	1–5	
Rurality			
Rural	11–15	1–5	0.95
Urban	439 (97.3)	105 (97.2)	
Ontario Marginalization (ON-Marg) Index summary score			
1-2	70 (15.5)	15 (13.9)	0.23
3	170 (37.7)	53 (49.1)	
4	167 (37.0)	31 (28.7)	
5	31 (6.9)	5 (4.6)	
Missing**	11–15	1–5	
Adjusted Charlson Comorbidity Index (CCI) score			
0	368 (81.6)	88 (81.5)	0.98
1+	83 (18.4)	20 (18.5)	
Tumour site			
Breast	46–50	1–5	0.33
Gastrointestinal	108 (23.9)	22 (20.4)	
Genitourinary	47 (10.4)	15 (13.9)	
Gynaecological	25 (5.5)	9 (8.3)	
Haematological	29 (6.4)	7 (6.5)	
Lung	73 (16.2)	19 (17.6)	
Other***	123 (27.3)	32 (29.6)	
Used chemotherapy in 30 days	139 (30.8)	27 (25.0)	0.23

prior to ESAS-r?			
Used radiotheapy in 30 days prior to ESAS-r?	**58 (12.9)**	**23 (21.3)**	**0.03**
Location of first PC visit			
Clinic	424 (94.0)	102 (94.4)	0.86
Other****	27 (6.0)	6 (5.6)	
Used home care?	105 (23.3)	27 (25.0)	0.71
Days from diagnosis to first PC interaction, median [IQR], days	**86 [197] (n = 432)**	**35 [99] (n = 106)**	**< 0.01**
Days from diagnosis to first ESAS-r report, median [IQR], days	**88 [202] (n = 432)**	**34 [105] (n = 106)**	**< 0.01**
Location of death			
Hospital	**149 (33.0)**	**66 (61.1)**	**< 0.01**
Non-hospital	**146 (32.4)**	**25 (23.1)**	
Alive or missing‡	**156 (34.6)**	**17 (15.7)**	
Survival, median [IQR], days	**107 [177] (n = 295)**	**36 [100] (n = 91)**	**< 0.01**
Survival†			
0-30	**46 (10.2)**	**43 (39.8)**	**< 0.01**
31-90	**83 (18.4)**	**17 (15.7)**	
91+	**166 (36.8)**	**31 (28.7)**	
Alive or missing‡	**156 (34.6)**	**17 (15.7)**	

* Q1: 29200–36400 CAD, Q2: 36400–42800 CAD, Q3: 42800–48400 CAD, Q4: 48400–66000 CAD ; ** Includes patients residing in postal codes too small to be assigned a marginalization score; *** Includes central nervous system, endocrine, head and neck, bone and soft tissue (sarcoma), skin, and unknown tumours; **** Includes inpatient hospital, home visit, and no first PC visit setting; † Note that patients did not necessarily die during their hospitalization; ‡ Either did not die during observation period, or death not captured in database.

Table S4. Multivariable binary logistic regression of ESAS-r symptom complexity and acute care utilization within 7 days.

**Characteristic (n = 559)**	**Emergency department visit or hospital admission within 7 days**	
	**aOR (95% CI)**	**p value**
Symptom complexity		
Low	Ref	
Medium	1.56 (0.60-4.05)	0.37
High	**2.83 (1.18-6.77)**	**0.02**
Age categories		
20-55	Ref	
56-65	1.32 (0.43-4.08)	0.63
66-75	0.72 (0.22-2.37)	0.59
76-85	1.07 (0.34-3.40)	0.91
86+	0.90 (0.25-3.21)	0.87
Sex		
Female	Ref	

Male	1.18 (0.62-2.23)	0.62
Ontario Marginalization (ON-Marg) Index summary score		
1-2	Ref	
3	0.81 (0.35-1.85)	0.61
4	0.46 (0.19-1.14)	0.09
5	0.57 (0.14-2.39)	0.44
Missing**	0.91 (0.17-5.00)	0.91
Adjusted Charlson Comorbidity Index (CCI) score		
0	Ref	
1+	1.52 (0.76-3.03)	0.24
Tumour site		
Breast	Ref	
Gastrointestinal	1.83 (0.45-7.43)	0.40
Genitourinary	1.51 (0.31-7.33)	0.61
Gynaecological	2.63 (0.51-13.45)	0.25
Haematological	1.32 (0.22-7.77)	0.76
Lung	2.69 (0.66-10.98)	0.17
Other***	1.78 (0.45-7.07)	0.42
Chemotherapy in 30 days prior to ESAS-r		
No	Ref	
Yes	**0.48 (0.23-0.98)**	**0.04**
Radiotherapy in 30 days prior to ESAS-r		
No	Ref	
Yes	1.33 (0.65-2.78)	0.44
Involvement of home care		
No	Ref	
Yes	0.89 (0.46-1.77)	0.76
Days from latest diagnosis to first PC interaction, median [IQR], days	**1.00 (1.00-1.00)**	**0.05**

** Includes patients residing in postal codes too small to be assigned a marginalization score; *** Includes central nervous system, endocrine, head and neck, bone and soft tissue (sarcoma), skin, and unknown tumours.

Table S5. Multivariable binary logistic regression of ESAS-r symptom complexity and acute care utilization within 14 days.

**Characteristic (n = 559)**	**Emergency department visit or hospital admission within 14 days**	
	**aOR (95% CI)**	**p value**
Symptom complexity		
Low	Ref	
Medium	0.99 (0.50-1.95)	0.98
High	1.78 (0.97-3.28)	0.07
Age categories		
20-55	Ref	

56-65	1.12 (0.46-2.71)	0.81
66-75	0.93 (0.37-2.31)	0.87
76-85	1.03 (0.42-2.55)	0.95
86+	0.62 (0.22-1.78)	0.38
Sex		
Female	Ref	
Male	1.54 (0.92-2.58)	0.10
Ontario Marginalization (ON-Marg) Index summary score		
1-2	Ref	
3	1.30 (0.65-2.60)	0.46
4	0.68 (0.32-1.44)	0.31
5	0.81 (0.25-2.62)	0.72
Missing**	1.46 (0.39-5.45)	0.57
Adjusted Charlson Comorbidity Index (CCI) score		
0	Ref	
1+	1.02 (0.57-1.82)	0.96
Tumour site		
Breast	Ref	
Gastrointestinal	1.59 (0.48-5.30)	0.45
Genitourinary	2.49 (0.67-8.88)	0.17
Gynaecological	**4.55 (1.21-17.04)**	**0.03**
Haematological	2.29 (0.56-9.32)	0.25
Lung	2.11 (0.62-7.14)	0.23
Other***	2.57 (0.80-8.24)	0.11
Chemotherapy in 30 days prior to ESAS-r		
No	Ref	
Yes	0.66 (0.39-1.11)	0.12
Radiotherapy in 30 days prior to ESAS-r		
No	Ref	
Yes	1.70 (0.95-3.04)	0.07
Involvement of home care		
No	Ref	
Yes	1.02 (0.60-1.72)	0.96
Days from latest diagnosis to first PC interaction, median [IQR], days	**1.00 (1.00-1.00)**	**< 0.01**

** Includes patients residing in postal codes too small to be assigned a marginalization score; *** Includes central nervous system, endocrine, head and neck, bone and soft tissue (sarcoma), skin, and unknown tumours.
